# Yield of HIV-associated tuberculosis during intensified case finding in resource-limited settings: a systematic review and meta-analysis

**DOI:** 10.1016/S1473-3099(09)70326-3

**Published:** 2010-02

**Authors:** Katharina Kranzer, Rein MGJ Houben, Judith R Glynn, Linda-Gail Bekker, Robin Wood, Stephen D Lawn

**Affiliations:** a The Desmond Tutu HIV Centre, Institute for Infectious Disease and Molecular Medicine, Faculty of Health Sciences, University of Cape Town, Cape Town, South Africa; b Clinical Research Unit, Department of Infectious and Tropical Diseases, London School of Hygiene and Tropical Medicine, London, UK; c Infectious Disease Epidemiology Unit, Department of Epidemiology and Public Health, London School of Hygiene and Tropical Medicine, London, UK

## Abstract

Intensified case finding is the regular screening for evidence of tuberculosis in people infected with HIV, at high risk of HIV, or living in congregate settings. We systematically reviewed studies of intensified case finding published between January, 1994, and April, 2009. In 78 eligible studies, the number of people with tuberculosis detected during intensified case finding varied substantially between countries and target groups of patients. Median prevalence of newly diagnosed tuberculosis was 0·7% in population-based surveys, 2·2% in contact-tracing studies, 2·3% in mines, 2·3% in programmes preventing mother-to-child transmission of HIV, 2·5% in prisons, 8·2% in medical and antiretroviral treatment clinics, and 8·5% in voluntary counselling and testing services. Metaregression analysis of studies that included only people with HIV showed that for each increment in national prevalence of tuberculosis of 100 cases per 100 000 population, intensified case finding identified an additional one case per 100 screened individuals (p=0·03). Microbiological sputum examination of all individuals without prior selection by symptom screening yielded an additional four cases per 100 individuals screened (p=0·05). Data on the use of serial screening, treatment outcomes in actively identified cases of tuberculosis, and cost-effectiveness, however, were lacking. Concerted action is needed to develop intensified case finding as an important method for control of tuberculosis.

## Introduction

The sixth Millennium Development Goal target of halving the 1990 prevalence of tuberculosis and death rates by 2015 will not be achieved if present trends continue.^1^ Any shortfall from this target worldwide will be associated with a failure to achieve these targets in sub-Saharan Africa where HIV has seriously undermined the control of tuberculosis. Worldwide there are more than 1·3 million cases of HIV-associated tuberculosis each year, resulting in almost half a million deaths; sub-Saharan Africa is estimated to account for 79% of this disease burden.^2^ Although the incidence of HIV-associated tuberculosis worldwide is estimated to have peaked in 2006,^2^ more progress is needed in reducing prevalence and mortality.

The WHO directly observed short-course strategy, which relies on a process of passive tuberculosis case finding, has helped to control tuberculosis in many parts of the world but not in countries with generalised epidemics of HIV (prevalence of HIV greater than 1% in the general population).^2^, ^3^ In recognition of this failure, WHO and the Stop TB Partnership developed an interim policy on collaborative tuberculosis and HIV activities^4^ that included additional interventions to reduce the burden of tuberculosis in people living with HIV. Within this policy the four core prevention strategies for tuberculosis were intensified case finding, isoniazid preventive therapy, tuberculosis infection control, and scale-up of antiretroviral therapy.^5^, ^6^

Despite clear policy guidelines, antiretroviral therapy is the only one of the four core strategies that has been used at a large scale.^6^ Use of isoniazid preventive therapy is still limited and provided for only 0·1% of individuals infected with HIV.^2^ Data on the use of tuberculosis infection control are scarce. Intensified case finding among patients attending HIV care services reached just 2·2% of the 33 million people estimated to be living with HIV in 2007.^2^ To address the substantial gap in the scale-up of these interventions, WHO launched the 3Is policy initiative in 2008, which is a three-pronged strategy of intensified case finding, isoniazid preventive therapy, and tuberculosis infection control to be used with the scale-up of antiretroviral therapy.^7^

Several terms have been used for the screening of patients for active tuberculosis. The terms intensive case finding, active case finding, and enhanced case finding are often used interchangeably and all refer to strategies to identify and treat people with tuberculosis who have not sought diagnostic services on their own initiative. However, whereas intensified case finding and active case finding require face-to-face contact and on-site screening, enhanced case finding works primarily through making populations aware of the symptoms of tuberculosis and encouraging self-presentation to medical services.^8^ In the 3Is policy, intensified case finding is defined as “the regular screening of all people with HIV or at high risk of HIV or in congregate settings (such as mines, prisons, military barracks) for symptoms and signs of TB followed promptly with diagnosis and treatment”, and this is the definition we have used in this Review.^7^ The policy also recommends screening of household contacts of people with tuberculosis.^7^

Intensified case finding is the central intervention of the 3Is strategy, because its aim is to identify patients as either having active tuberculosis (and in need of treatment) or free of the disease (and warranting preventive therapy), although in practice the status of some patients will be unclear and these patients need prospective evaluation. Intensified case finding is also the key means by which the prevalence of untreated disease can be reduced within clinical services, congregate settings, and in the community, thereby reducing the transmission of tuberculosis. In view of this new policy initiative, there is a great need for data that inform development of strategies of intensified case finding.

Whereas the specific investigations (eg, sputum microscopy, sputum culture, chest radiography) used in screening for tuberculosis might have an important affect on the yield of new cases of tuberculosis identified,^9^ overall investigational strategy and target group are also very important. For example, establishing that a patient has the symptoms of tuberculosis has traditionally been central to screening strategies, and yet recent studies^9^, ^10^, ^11^, ^12^, ^13^ have shown that a proportion of patients with HIV-associated tuberculosis have asymptomatic disease or very minor symptoms. As a result, the yield of intensified case finding in some groups might be higher if all individuals are investigated without preselection.

The identification of groups for which intensified case finding should be prioritised is a further issue. The yield of cases of tuberculosis is likely to be highly variable, depending on a range of factors that include the local prevalence of tuberculosis, the function of local tuberculosis control services, the prevalence of HIV in the target group, and the degree of associated immunodeficiency. In turn, the prevalence of tuberculosis detected established the number needed to screen (NNS) to identify one new case of undiagnosed active tuberculosis. Other important factors affecting decisions on implementing intensified case finding include the feasibility and cost of the screening, the laboratory capacity, and treatment outcomes in newly detected cases. Treatment outcomes are particularly important because adherence might be lower in patients identified in active screening without preselection than in those detected passively, potentially limiting the effect of intensified case finding on the control of tuberculosis.

The purpose of this systematic review is to examine strategies of intensified case finding as outlined in the WHO 3Is policy.^7^ Key outcomes of interest in reviewed published work include the prevalence of tuberculosis in specifically targeted groups, the associated NNS, the screening strategy used, cost-effectiveness, and treatment outcomes of newly detected cases.

## Methods

### Search strategy and selection criteria

The searches and review process were done according to a prespecified protocol. We aimed to identify studies using intensified case finding in resource-limited settings. Both cross-sectional and cohort studies published in all languages were eligible for inclusion. We searched available systematic and narrative reviews for active case finding and household-contact investigations. Two systematic reviews were identified.^8^, ^14^ Additionally, we searched Medline, EmBase, and Global Health for reports published through April, 2009, and African Health Line up to March, 2009. Search strategies are presented in the webappendix. The search strategy for African Health Line included “tuberculosis”, “active or intensified or enhanced case finding”, “prisons or prisoners”, “mines or miners”, “homosexuals”, “voluntary counselling”, and “testing or VCT”. Abstract books covering the years 1998–2008 of the World Conference on Lung Health published by the International Union Against Tuberculosis and Lung Disease were hand searched. Reference lists of primary studies, reviews, and editorials identified by the above methods were hand searched. Experts in the specialty were contacted for additional publications.

According to the WHO 3Is policy, regular screening for tuberculosis is recommended in groups at high risk of infection with HIV, individuals infected with HIV, groups living in congregate settings, and in contacts of people with tuberculosis. The strategy to be used in contact tracing is not explicitly described in the policy, and so for the purposes of this Review we focused on contact tracing studies in populations where the prevalence of HIV was at least 5% among notified cases of tuberculosis. We also defined groups at high risk for infection with HIV as prisoners, commercial sex workers, injecting drug users, men who have sex with men, and individuals attending voluntary counselling and testing centres and sexually transmitted disease clinics. Even though the 3Is policy does not recommend mass screening on a population level, it is arguable that general populations in sub-Saharan Africa constitute groups at high risk for HIV. Thus, surveys of the prevalence of tuberculosis in populations with a prevalence of HIV of greater than 5% were included in this Review.

Our Review was limited to studies published from 1993 (when WHO declared tuberculosis to be a worldwide emergency) to 2009.^15^ We only included studies in low-income and middle-income countries, as defined by the World Bank in 2008,^16^ and studies that screened a minimum of 100 people. We excluded editorials, case studies, case reports, studies screening only children younger than 15 years, and studies with unclear screening strategies or denominators. However, we included contact-tracing studies that screened both adults and children.

The initial database created from the electronic searches was compiled, duplicate citations were eliminated, and citations were screened by title and abstract to capture potentially relevant studies. The full text of these studies was obtained and reviewed according to inclusion and exclusion criteria. Screening of full text from citations found to be potentially relevant was done by two reviewers (KK and SDL). Queries on study design and study quality were discussed with another reviewer (JRG), and studies were only included if there was consensus.

### Data extraction and analysis

Where data from a single study were present in multiple publications, these data were compiled. The data extraction form was adapted from the STROBE statement checklist.^17^ Variables recorded included title, objective, year of study, year of publication, study design, setting, eligibility criteria of participants, sources and methods of selection of participants, screening strategy, diagnostic criteria for tuberculosis, internal and external quality checks, efforts to reduce bias, numbers of individuals at each stage of the study, number of cases of tuberculosis, prevalence of tuberculosis, and funding source. One reviewer (KK) extracted data from all eligible studies. A second reviewer (SDL) independently extracted data from 20 of the included studies. The ratings given by the two reviewers were in complete agreement.

Primary outcome of interest was the yield of intensified case finding as defined by the prevalence of newly microbiologically confirmed or clinically or radiologically diagnosed cases of tuberculosis in the target population and the NNS to find one new case of tuberculosis. The NNS was calculated as the reciprocal of the prevalence of newly diagnosed tuberculosis. Secondary outcomes of interest were the screening strategy used, treatment outcomes, and cost per case found.

All analyses were done in Stata 10. We report medians and ranges; weighted medians were used to compare yield of screening for tuberculosis in individuals infected with HIV by use of different screening strategies. We used inverse-variance-weighted metaregression to investigate the association between yield of intensified case finding and country prevalence of tuberculosis, prevalence of HIV in the general population, screening strategies, availability of culture, and HIV status of the study population.^18^, ^19^ Country prevalence estimates for tuberculosis and HIV were obtained from the WHO Global Health Atlas.^20^ We analysed residuals to check model assumptions.

## Results

**Figure 1:**
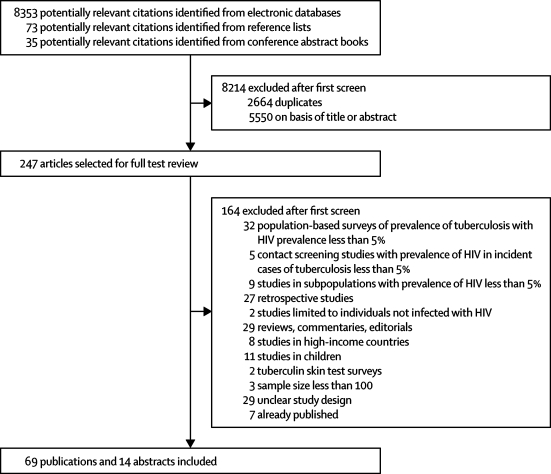
Study selection process

83 publications (69 published papers and 14 abstracts) published between January, 1994, and April, 2009, were eligible for inclusion (figure 1). After compiling multiple publications of the same study a total of 78 studies were included. Most studies (55) were published in the past 6 years, with the largest numbers of publications being in 2008 (12) and 2007 (13).

Of those studies included, 30 were from congregate settings, ten from voluntary counselling and testing settings, three from programmes preventing mother-to-child transmission (PMTCT), 16 from antiretroviral therapy and medical clinics, ten contact tracing studies, eight population-based surveys of the prevalence of tuberculosis, and one study among men who have sex with men and injecting drug users. Most studies were from sub-Saharan Africa (41); others were from the Americas (22) and Asia (19).

The proportion of eligible individuals who agreed to participate in these studies was variable, ranging from 40–100% in prisons, 44–100% in voluntary counselling and testing and PMTCT settings, 35–100% in clinic-based settings, 83–100% in contact tracing studies, and 66–100% in population-based surveys (webappendix). Clear external and internal quality-control procedures for microbiological and radiological investigations were only recorded in three studies in congregate settings, two studies in clinic-based settings, one study in household contacts, and four population-based surveys.

The prevalence of newly diagnosed tuberculosis varied greatly between different countries and specific target populations (table 1). The minimum was 0·01% in a contact-tracing study from Peru and the maximum was 24·7% in an antiretroviral therapy clinic in South Africa. Median NNS varied greatly, ranging from 148 in population-based prevalence surveys to just 12 in voluntary counselling and testing services and in antiretroviral therapy and medical clinics (table 1).

**Table 1:** Tuberculosis prevalence and the number needed to screen to identify one new case in different target groups

	**Number of studies**	**Regions represented**	**Median prevalence of newly diagnosed tuberculosis (range)**	**Median number needed to screen (range)**
Congregate settings (all)^21^, ^22^, ^23^, ^24^, ^25^, ^26^, ^27^, ^28^, ^29^, ^30^, ^31^, ^32^, ^33^, ^34^, ^35^, ^36^, ^37^, ^38^, ^39^, ^40^, ^41^, ^42^, ^43^, ^44^, ^45^, ^46^, ^47^, ^48^, ^49^, ^50^	30	Africa, Asia, the Americas	2·2% (0·1–7·2)	45 (14–833)
Congregate settings (prisons)^21^, ^22^, ^23^, ^24^, ^25^, ^26^, ^27^, ^28^, ^29^, ^30^, ^31^, ^32^, ^33^, ^34^, ^35^, ^36^, ^37^, ^38^, ^39^, ^40^, ^41^	21	Africa, Asia, the Americas	2·5% (0·1–7·2)	40 (14–833)
Congregate settings (prisons)^27^, ^28^, ^29^, ^30^, ^31^, ^32^, ^33^	7	Sub-Saharan Africa	3·6% (1·8–7·2)	28 (14–55)
Congregate settings (mines)^43^, ^44^, ^45^, ^46^, ^47^, ^48^	6	Africa, Asia	2·3% (1·2–5·0)	43 (20–86)
Voluntary counselling and testing services^51^, ^52^, ^53^, ^54^, ^55^, ^56^, ^57^, ^58^, ^59^, ^60^, ^61^	10	Africa, Asia, the Americas	8·5% (0·8–23·6)	12 (4–123)
Prevention of mother-to-child transmission services^62^, ^63^, ^64^	3	Africa, Asia	2·3% (2·1–3·5)	44 (29–47)
Antiretroviral therapy and medical clinics^9^, ^10^, ^65^, ^66^, ^67^, ^68^, ^69^, ^70^, ^71^, ^72^, ^73^, ^74^, ^75^, ^76^, ^77^, ^78^, ^79^, ^80^, ^81^	16	Africa, Asia, the Americas	8·2% (1·4–24·7)	12 (4–71)
Antiretroviral therapy and medical clinics^9^, ^10^, ^70^, ^71^, ^72^, ^73^, ^74^, ^75^, ^76^, ^77^	8	Sub-Saharan Africa	8·6% (3·6–24·7)	12 (4–28)
Contact tracing^82^, ^83^, ^84^, ^85^, ^86^, ^87^, ^88^, ^89^, ^90^, ^91^	10	Africa, Asia, the Americas	2·2% (0·01–14·5)	45 (7–10 000)
Population-based surveys^11^, ^92^, ^93^, ^94^, ^95^, ^96^, ^97^, ^98^, ^99^	8	Sub-Saharan Africa	0·7% (0·02–3·5)	148 (29–5000)

Results of studies of intensified case finding in prisons, psychiatric hospitals, mines, and refugee camps are presented in the webappendix. HIV status was established in three of 30 studies and screening strategies varied substantially, with most using an initial questionnaire on the symptoms of tuberculosis followed by diagnostic testing of people suspected to have tuberculosis. Three of six studies in miners combined the screening of symptoms with chest radiology to define those people suspected of having tuberculosis, whereas the other three studies used sputum microbiology irrespective of symptoms.

The median NNS in all prisons was 40 (range 14–833), but was lower in studies in prisons in sub-Saharan Africa (median 28, range 14–55; table 1). The prevalence of HIV was established in only one study in a Brazilian prison (25%).^41^

Studies in miners and ex-miners found a median NNS of 43 (range 20–86; table 1). In one study enrolling only miners infected with HIV, the prevalence of newly diagnosed tuberculosis was 4·9%.^46^ Of the remaining five studies only one study tested miners for HIV, and reported a prevalence of 27%.^47^

Most studies in voluntary counselling and testing clinics, PMTCT programmes, and in groups at high risk of infection with HIV (men who have sex with men and injecting drug users)^100^ identified people suspected of having tuberculosis by screening their symptoms before using diagnostic tests (webappendix). Sputum microscopy was the only microbiological test available in half of the studies. The median NNS was 12 (range 4–123) in voluntary counselling and testing settings and 44 (range 29–47) in PMTCT settings (table 1).

Intensified case finding was done as part of isoniazid preventive therapy programmes or before antiretroviral therapy was started, in individuals infected with HIV accessing health care or enrolled in home-based care services. Half of the studies used questionnaires on the symptoms of tuberculosis as the first step before microbiological investigations for symptomatic individuals (webappendix). Median NNS was 12 (range 4–71; table 1).

Results from contact-tracing studies (webappendix) are not directly comparable with results from other settings since most studies included children. Microbiological examinations were only done in individuals that were symptomatic or in individuals with positive tuberculin skin tests. Median NNS was 48 (range 7–10 000; table 1).

Five of eight population-based surveys of the prevalence of tuberculosis in settings with high prevalence of HIV used a step-wise screening approach with screening for the symptom of tuberculosis preceding microbiological examination. The remaining three studies examined the sputum in all individuals irrespective of symptoms (webappendix). Median NNS was 148 (range 29–5000; table 1).

**Figure 2:**
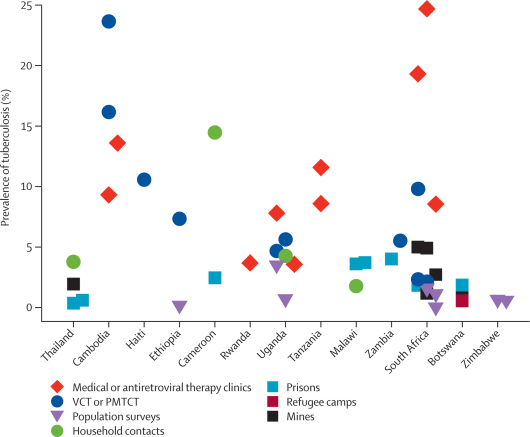
Prevalence of tuberculosis among individuals screened in different settings in countries with generalised epidemics of HIV VCT=voluntary counselling and testing. PMTCT=prevention of mother-to-child transmission.

Figure 2 summarises data from 47 studies in countries with generalised epidemics of HIV. More than half the studies (27) reported a prevalence of newly diagnosed tuberculosis in the screened population of greater than 3% (NNS less than 33). Excluding population surveys, two-thirds (26 of 39) of the studies reported prevalence of newly diagnosed tuberculosis of greater than 3%.

The screening strategies varied widely across the studies. Symptom screening was used in all but one of the prison studies, screening of mine workers invariably included chest radiography, and all contact-screening studies used symptom screening and assessment of responses to the tuberculin skin test. Symptom questionnaires were diverse, ranging from any kind of respiratory symptoms to various durations of productive cough plus or minus weight loss, night sweats, fatigue, fever, and haemoptysis. Similarly, the number and timing of sputum samples varied substantially.

In 12 studies that included only individuals infected with HIV, sputum examination was done irrespective of symptoms. Nine of these studies were done in antiretroviral therapy services, medical clinics, or home-based HIV care programmes and the remaining three were done in voluntary counselling and testing centres. All but one of these studies did both smears and cultures. 17 studies screened individuals infected with HIV with symptom-based questionnaires preceding sputum examination of people suspected to have tuberculosis. These studies were done in antiretroviral therapy and medical clinics (seven studies), voluntary counselling and testing centres (seven), and PMTCT programmes (three). Of these 17 studies, seven only did sputum smears whereas ten did both smears and cultures. In the subset of studies in which both smears and cultures were done, the prevalence of newly diagnosed tuberculosis in individuals infected with HIV was slightly lower (weighted median 9·8%) in studies only investigating preselected people suspected to have tuberculosis on the basis of symptom screening compared with studies doing sputum smears and cultures on everyone irrespective of symptoms (weighted median 11·6%).

Because the strategies and yield of intensified case finding were very heterogeneous between studies done in different countries and patient groups, we sought to identify variables independently associated with the yield of tuberculosis by use of metaregression analysis (Table 2, Table 3).

**Table 2:** Univariate analysis of factors potentially affecting yield of intensified case screening in populations with mixed or unknown HIV status

	**Slope (95% CI)**	**p value**
Country prevalence of tuberculosis*	−0·2 (−0·5 to 0·1)	0·21
Country prevalence of HIV†	−4·8 (−12·8 to 3·3)	0·24
Availability of culture‡	−0·3 (−1·6 to 1·1)	0·65
Symptom screening§	−0·3 (−2·4 to 1·8)	0·77

* One unit increase=100 cases of tuberculosis per 100 000 people.

† One unit increase=10% increase in prevalence.

‡ Coded as 0 if no culture available or 1 if culture available.

§ Coded as 0 if symptom screening used to identify people suspected to have tuberculosis or 1 if all individuals screened with sputum smears or culture.

**Table 3:** Factors potentially affecting yield of intensified case screening in populations with individuals infected with HIV

	**Slope (95% CI) in univariate analysis**	**p value in univariate analysis**	**Slope (95% CI) in multivariate analysis**	**p value in multivariate analysis**
Country prevalence of tuberculosis*	1·1 (0·2 to 2·0)	0·02	1 (0·1 to 1·9)	0·03
Country prevalence of HIV†	1·3 (−17·5 to 43·1)	0·4	..	..
Availability of culture‡	2·0 (−2·7 to 6·7)	0·39	..	..
Symptom screening§	4·3 (0·34 to 8·2)	0·03	3·7 (0·05 to 7·4)	0·05

* One unit increase=100 cases of tuberculosis per 100 000 people.

† One unit increase=10% increase in prevalence.

‡ Coded as 0 if no culture available or 1 if culture available.

§ Coded as 0 if symptom screening used to identify people suspected to have tuberculosis or 1 if all individuals screened with sputum smears or culture.

Of the 78 studies included in the analysis, some target populations included only individuals infected with HIV (30 studies), whereas the remainder included individuals with and without HIV (congregate settings, contact screening, and population-based prevalence studies). Metaregression analysis was therefore stratified with regards to HIV status of the screened population.

In the univariate analysis of studies with patients with mixed or unknown HIV-status (48 studies), national prevalence of tuberculosis and HIV, screening strategy of microbiological investigations in all individuals, and availability of culture were not associated with yield of screening (table 2).

By contrast, univariate analysis of studies that only included individuals infected with HIV (30 studies) found that both the use of symptom prescreening and the country prevalence of tuberculosis were associated with the detected yield of tuberculosis (table 3). However, the availability of culture and the national prevalence of HIV were not associated with the detected yield of tuberculosis. Multivariate analysis showed that microbiological sputum examination (smear or culture) on all individuals without prior selection on the basis of screening for symptoms detected an additional four cases per 100 individuals screened (p=0·05). Furthermore, an increment in country prevalence of tuberculosis of 100 cases of tuberculosis per 100 000 population was associated with an additional yield of one case per 100 individuals screened (p=0·03).

Restricting the analysis to studies in individuals infected with HIV routinely doing both sputum smears and cultures as part of the microbiological investigations (22 studies) showed similar results with regards to effect estimate for symptom screening (slope 3·6, 95% CI −0·9 to 8·0).

Estimates of national prevalence of tuberculosis are infrequently on the basis of prevalence survey data, they are instead derived from estimates of the incidence of tuberculosis. In view of this and that national estimates of incidence of tuberculosis are more readily available, we repeated the metaregression analysis with the inclusion of estimated incidence of tuberculosis rather than estimated prevalence. In this model, an increment in country incidence of tuberculosis of 100 cases of tuberculosis per 100 000 people was associated with an additional yield of 0·7 case per 100 individuals screened (p=0·04).

Only three studies reported treatment outcomes of individuals diagnosed with tuberculosis during intensified case finding. In a population-based study in South Africa, only 13 (56%) of 23 people actively detected with tuberculosis completed treatment.^97^ Among 24 women infected with HIV in India diagnosed with tuberculosis after giving birth, 21 started treatment for tuberculosis and 17 (70%) were either cured or still receiving treatment at time of analysis.^62^ In the Côte d'Ivoire, of 134 prisoners diagnosed with tuberculosis, 99 (74%) were cured, 32 (24%) died, and in three (2%) treatment failed.^28^ None of these studies had data for treatment outcome available for those diagnosed passively.

None of the studies included in this systematic review did costing or cost-effectiveness analyses.

## Discussion

Intensified case finding is a key component of the WHO 3Is policy that aims to strengthen the public health response to the epidemic of HIV-associated tuberculosis—a major stumbling block to attainment of the sixth Millennium Development Goal targets for worldwide control of tuberculosis. In this systematic review, we included 78 studies from 27 different countries, most from sub-Saharan Africa. The yield of screening for tuberculosis varied greatly between countries and target populations, with the highest yields in antiretroviral therapy and voluntary counselling and testing clinics, and the lowest yields in population-based surveys of the prevalence of tuberculosis. Our analysis showed that the yield of intensified case finding depends strongly on the prevalence of HIV of the target population, the national prevalence of tuberculosis, and the screening strategy. In studies only screening individuals infected with HIV, each increment in national prevalence of tuberculosis of 100 cases per 100 000 people resulted in an additional yield of one case per 100 individuals screened. Furthermore, substantially higher yields can be achieved if all individuals have sputum examination irrespective of symptoms. These data will help inform implementation of intensified case finding policies.

Using a process of passive case finding, the directly observed short-course strategy has failed to control rising incidence rates of HIV-associated tuberculosis in resource-limited settings,^101^ and many patients dying with HIV/AIDS have tuberculosis that has not been diagnosed.^102^ Intensified case finding aims to increase case-detection rates and shorten the time to diagnosis of tuberculosis, thereby reducing morbidity and mortality and shortening the period of infectiousness, and it should, in turn, reduce the risk of transmitting tuberculosis in community and health-care settings.^103^, ^104^, ^105^, ^106^ However, intensified case finding is more resource-intensive than passive case finding and a number of key issues must be considered. These issues include the populations to be targeted, the NNS to identify each new case, the screening strategy, laboratory capacity, operational feasibility, outcomes of newly identified cases of tuberculosis, and costs.

Prevalence of newly diagnosed tuberculosis and subsequently the NNS varied widely between different target populations with a median of 148 (range 29–5000) in population-based surveys, 45 (7–10 000) in contact tracing studies, 43 (20–86) in mines, 40 (14–833) in prisons, 12 (4–123) in voluntary counselling and testing, 44 (29–47) in PMTCT settings, and 12 (4–71) in antiretroviral therapy or medical clinics.

The upper limit for the NNS at which intensified case finding is deemed useful might differ between target populations. For example, the importance of identification of tuberculosis among prisoners is high because of the high probability of transmission to other prisoners, and among pregnant women it is high in view of the probability of transmission to unborn children.^107^, ^108^ Intensified case finding might also be prioritised in settings with known high prevalence of drug-resistant tuberculosis, such as prisons.

Guidelines originating from the era before HIV assume that state programmes for tuberculosis in resource-limited settings should screen an average of ten people suspected to be infected to identify one smear-positive case to prevent laboratory overload.^109^ Laboratory equipment and consumables for tuberculosis are allocated on this basis. More recent experience has shown that in practice an average of seven people newly suspected of having tuberculosis are screened to identify one sputum smear-positive case of tuberculosis, although this number varies widely (3·3–20).^110^ Irrespective of the specific target populations, we found that intensified case finding will need more resources. Scale-up will only be possible if laboratory capacity increases in parallel and quality assurance procedures are adequate.

The effectiveness of intensified case finding depends on the screening strategy used. In studies of groups of patients infected with HIV, intensified case finding with microbiological (sputum smear or culture) investigation in all patients irrespective of symptoms detected an additional four cases per 100 individuals screened. This finding is consistent with the observation that a substantial proportion of individuals infected with HIV have subclinical tuberculosis when screened actively.^10^, ^11^, ^12^, ^13^, ^96^

The WHO 3Is strategy recommends repeated intensified case finding in individuals infected with HIV. However, no empirical data exist on the use of this type of serial screening, especially in a population of people infected with HIV. Serial screening with mass radiography in Czechoslovakia in 1961–72 showed a decrease in prevalence of newly diagnosed tuberculosis from 36 cases per 100 000 people to 18 cases per 100 000 people.^111^ Thus, a similar effect might happen with serial intensified case finding in individuals infected with HIV, although the yield in groups of patients infected with HIV is also likely to vary in parallel with changes to the degree of immunodeficiency. Studies establishing the yield obtained during serial screening, the optimum screening interval, and the implications for laboratory capacity are needed.

The diagnosis of tuberculosis by active screening is typically less advanced than the diagnosis of disease during passive case finding.^105^, ^112^, ^113^ Patients with less advanced disease might, in turn, have lower numbers of adherence to treatment, potentially resulting in poorer treatment outcomes among those whose disease is detected by intensified case finding. However, data on treatment outcomes are scarce. A study from South Africa found that the rates of treatment for tuberculosis were low (73%) among actively detected cases.^112^ Similar results were reported from population-based prevalence surveys from India and Nepal showing higher treatment refusal and default rates in actively compared with passively detected cases of tuberculosis.^114^, ^115^ Therefore, intensified case finding must be accompanied with effective means of ensuring that newly identified cases of tuberculosis are effectively treated.

Our metaregression analysis found that each increment in national prevalence of tuberculosis of 100 cases per 100 000 people was associated with an additional yield of about one case per 100 screened individuals infected with HIV. These data provide some basis for estimating the potential benefits of intensified case finding in different countries. Although none of the studies presented in this Review investigated cost-effectiveness, modelling suggests that population-based intensified case finding symptom screening every 7 years would be highly cost-effective in sub-Saharan Africa, averting 29 million cases of tuberculosis and 13 million deaths by 2050.^116^ One study from South Africa estimated a cost of US$12 for each person screened for tuberculosis in the community health centre and $7 per person in the primary health-care clinic. The cost per case of tuberculosis prevented was estimated to be $323–366, assuming that 25 cases of tuberculosis would be prevented for every 100 cases of tuberculosis detected by intensified case finding.^117^ However, more country-specific and setting-specific data on costs are needed to inform policy makers.

Our systematic review has several strengths. Publication bias was limited by the use of a prespecified comprehensive search strategy and review process that included published and unpublished studies without language restrictions. Reproducibility of data extraction was verified for a subset of studies. Heterogeneity of study results was investigated using metaregression analysis. This Review, which is primarily descriptive, also has limitations. Assessment of ascertainment bias at study level was limited because of the lack of reporting of internal and external quality-control procedures. Furthermore, variable uptake in the proportions of eligible individuals who agreed to participate in these studies might have influenced reported prevalence of tuberculosis. Regression analyses could be subject to residual confounding.

## Conclusion

In countries with high prevalence of tuberculosis, intensified case finding among individuals infected with HIV identifies a high yield of people with tuberculosis and the yield is significantly increased if all such individuals are screened microbiologically without preselection on the basis of the screening of symptoms. The data presented might help the prioritisation of specific groups for intensified case finding. Scaling-up of intensified case finding will need development of standardised screening algorithms, substantial increases in the capacities of quality-assured laboratories, and efficient systems to ensure that people newly diagnosed with tuberculosis receive adequate treatment. Concerted action in tandem with further research might help develop this policy as an important method to accelerate progress towards the tuberculosis targets within the sixth Millennium Development Goal.

## Search strategy and selection criteria

These are described in detail in the Methods section

## Web Extra Material

Supplementary webappendix
