# Supplementary material for: Yield of HIV-associated tuberculosis during intensified case finding in resource-limited settings: a systematic review and meta-analysis
Source: Lancet Infect Dis. 2010 Feb;10(2):93–102. doi: 10.1016/S1473-3099(09)70326-3 (PMC3136203; doi:10.1016/S1473-3099(09)70326-3)
Supplement: Supplementary webappendix [file mmc1.pdf]

THE LANCET Infectious Diseases

Supplementary webappendix

This webappendix formed part of the original submission and has been peer reviewed. We post it as supplied by the authors.

Supplement to: Katharina Kranzer, Rein M G J Houben, Judith R Glynn, Linda-Gail Bekker, Robin Wood, Stephen D Lawn. Yield of HIV-associated tuberculosis during intensified case finding in resource-limited settings: a systematic review and meta-analysis. *Lancet Infect Dis* 2010; **10:** 93–102.

Search strategy

SET		Medline	Embase	Global Health
1	tuberculosis		tuberculosis	
2			TUBERCULOSIS	
3			Set 1 and 2 were combined with "or"	
4	screening		screening	
5			MASS SCREENING	
6			(active or enhanced or intensified) adj1 (case adj1 finding)	
7			CONTACT TRACING	
8			(household adj1 contact*) or (contact adj1 tracing)	
9			Set 4-8 were combined with "or "	
10	Isoniazid		ISONIAZID	
11			isoniazid	
12			(isoniazid adj1 preventive adj1 therapy) or ipt	
13	HIV/AIDS	AIDS-RELATED OPPOTUNISTIC INFECTION, PREVENTION	AIDS	AIDS
14			Set 10-13 were combined with "or"	
15	subgroups at high risks of Hiv infection or in congregated settings		PRISONS or PRISONERS	
16			inmate* or prison*	
17			MINING	
18			miner*	
19			REFUGEES	
20			refugee* or (internally adj1 displaced)	
21		HOSPITALS, PSYCHIATRIC	psychiatric adj1 hospital	psychiatric adj1 hospital
22		SUBSTACE ABUSE, INTRAVENOUS	INTRAVENOUS DRUG ABUSE	INJECTING DRUG ABUSE
23		(injecting adj1 drug adj1 user) or ivdu	(injecting adj1 drug adj1 user) or ivdu	(injecting adj1 drug adj1 user) or ivdu
24			PROSTITUTION	
25			prostitute* or csw or (commercial adj1 sex) or prostitution	
26			HOMOSEXUALITY, MALE	
27			msm or gay or homosexual*	
28			vct or (voluntary adj1 counselling adj1 testing)	
29			Set 15-28 were combined with "or"	
30			Set 29, 14 and 9 were combined with "or"	
31			Set 30 and 3 were combined with "and"	
32			set 31 was limited to years "1994-2009"	
33			set 32 and (search strategy "country")[1] were combined with "and"	

Words written in capital letters were used as MeSH headings, the others were used as free text. Search terms which were the same for all databases are only mentioned once under the EMBASE heading

[1] search strategy "country" combined all midlle and low income countries (MeSH heading and free text) with or

Congregate settings

Author	Year of Publication	Year of study	Country	N eligible	N screened	Symptom screen	Exam	TST	CXR	antibiotics	S	C	TB prevalence	Comments prevalence
Prisons and correctional facilities														
Aerts[22]	2000	1997-98	Georgia	7630	7473*	1			(2)		2(MMM)	3	5.99%	445 S+/C+; 3 S-/C+
Salek[23]	2001	1999	Iran	2212	2212	1	2		2		2		0.68%	10 S+; 5 clin
Askarian[24]	2001	1997	Iran	319	319			1 (10)	2		3	4	2.50%	4 S+/C+/CXR+; 4 S-/C-/CXR+
Yazdanpana[25]	1999		Iran	1634	1634		1		2		2		0.12%	1 S+; 1 clin
Rao[26]	2004	2002	Pakistan	4870	4870	1			2		2(SSS)		0.66%	1 S+/CXR+; 31 S-/CXR+
Shah[27]	2003	2002	Pakistan	386	386	1					2(SMS)		3.89%	9 S+; 6 clin
Noeske[28]	2006	2003-04	Cameroon	2830	2474*	1				3	2(MM)	4(MM)	2.43%	60 S+ or C+
Koffi[29]	1997	1990-02	Ivory Coast	1861	1861	1	1		3		2		7.20%	108 S+; 26 S-/CXR+
Wang[30]	2003	2002	Botswana	1173	1027*	1			(2)		2(XXX)	2(XX)	1.85%	8S+; 11 S-
Banerjee[31]	2000	1997	Malawi	275	111*	1	1				2(XXX)		3.60%	4 S+
Nyangulu[32]	1997	1996	Malawi	1315	900*	1			4	3	2(SMS)		3.70%	18 S+;15 S-/clin
Habeenzu[33 a]	2007	2000-01	Zambia	6118*	1069	(1)					2(MMM)	2(MMM)	4.00%	168 S+/C+; 77 S-/C+
Pillay[34]	2003		South Africa	7341	7341	1					2	(2)	1.82%	
Naranbat[35]	2001		Mongolia	4811	4021*				1		2		2.04%	
Jittimanee[36]	2007	2004-05	Thailand	71594	71594	1					2(SSS)		0.35%	254 S+
Sretrirutchai[37]	2002	1998	Thailand	4751	4751	1			2		3	3	0.57%	27 S+/CXR+
Sanchez[38]	2007	2005	Brazil	1377	1372*				1		2(XX)	2(XX)	3.86%	36 S+/C+; 17 S-/C+
Abrahao[39]	2006	2000-01	Brazil	1122	1052*	1[a]		1[b](5)			2(X or XX)	2(X or XX)	2%[c]	8 S+/C+; 13 S-/C+
Fournet[40]	2006	2003	Brazil	789	581*	1			1		2(XX)	2(XX)	5.50%	17 S+; 12 S-/C+; 3 clin
Sanchez[41]	2005	2002	Brazil	1171	1052*	1			1		2(XX)	2(XX)	4.09%	14 S+; 14 S-/C+; 15 clin
Ferreira[42]	1996	1992-93	Brazil	378	350*	1	1		2		1(XX)	1(XX)	5.71%	2 S+; 15 S-/C+; 3 clin
Psychiatric hospitals														
Van Duc[43]	2008	2005	Vietnam	300	300	1			1		2(XX)		0.33%	1 S+
Mines														
Steen[44 d]	1997	1994	Botswana	303	303		1		1		2		1.32%	4 S+
Fielding[45]	2008	2006-07	South Africa	13482	13482	1			1		2(X)	2(X)	1.16%	
Girdler-Brown[46 d]	2008	1999	South Africa	779	624*	1			1		2(XXX)	2(XXX)	4.97%	18 S+ or C+; 13 clin
Day[47 e]	2006	1999-01	South Africa	1310	899*	1	1		1		1(SS)	1(SS)	4.89%	35 C+; 4 C-/cli, 5 EPTB
Corbett[48]	2004	2000-01	South Africa	2248	1734*	1			1		1(XX)	1(XX)	2.70%	9 S+/C+; 36 S-/C+; 2 clin
Aungkasuvapala[49]	1995	1995	Thailand	676	676		1		1		1(XXX)		1.92%	8 S+/CXR+; 5 S-/CXR+

Refugees and internally displaces														
Weinstock[50]	2001	1999	Georgia	4000	931*	1		1 (10)	2		3(MMM)	3(MMM)	0.54%	2 C+; 1 C-/CXR+; 2 C?/CXR+
Nelson[51]	2003	2002-03	Botswana	2777	1615*	1			3[f]		2(XXX)		0.25%	

[a]symptomatic prisoners were asked to present for screening, [b]asymptomatic prisoners had 1 smear and culture symptomatic prisoners and prisoners with positive TST had 2 smears and cultures, [c]3 of the active TB cases were on TB treatment, [d]studies in ex-miners, [e]study of HIV+ miners attending an HIV clinic, [f]CXR were performed for those unable to produce sputum

VCT, PMTCT, high risk groups

Author	Year of Publication	Year of study	Country	N(HIV+) eligible	N(HIV+) screened	N(HIV-) eligible	N(HIV-) screened	Symptom screen	Exam	TST	CXR	antibiotics	S	C	TB prevalence	Comments prevalence
VCT																
Shetty[52]	2008	2003-04	India	4479	4479	5142	5142	1					2		HIV+ 0.81%<br>HIV- 0.91%	51 S+; 20 S-; 12 EPTB
Shah[53]	2009	2005-06	Ethiopia	453	438*			1	1		1		1(SMS)	1(SMS)	7.31%	9 S+/C+; 18 S-/C+, 5 S+/C-
Mugisha[54]	2006	2001--03	Uganda	7453	6305*			1	1		4	3	2		4.65%	190 S+; 91 S-; 12 EPTB
Aisu[55]	1995	1991-92	Uganda	1524*	1424			1			2		2		5.60%	
Godfrey-Faussett[56]	1995		Zambia	181	90*			1	1		1		2	2	5.50%	
Naidoo[57]	2002	2000-01	South Africa	2261	1126*			1			2		2	2	9.77%	35 S+, 59 S+/C+, 39 clin PTB, 21 EPTB
Kanara, MMW[58, 59]	2008/2005	2001-03	Cambodia	1979	876*			1	1		(1)		1		23.63%	
Chheng[60]	2008	2005	Cambodia		124[a]		372[a]						1(SMS)	1(SMS)	HIV+ 16.13%<br>HIV- 2.42%	19 S+; 10 C+
Espinal[61]	1995	1991-93	Dom. Rep.	350	200*	364	200*	1	1	1 (5)	2		2(XXX)		HIV+ 14.50%<br>HIV- 5.00%	29 S+; 4 S-/clin; 6 EPTB
Burgess[62]	2001	1997	Haiti	474	474	853	853	1	1		2		2(SMM)	2(SMM)	HIV+ 10.55%<br>HIV- 3.05%	HIV+ 11 S+/C+; 14 S-/C+; 25 S-/C-<br>HIV- 17 S+/C+; 9 S-/C-
PMTCT or postpartum care																
Gupta[63 b]	2007	2002-05	India	715	688*			1		1 (5)		2	2	2	3.49%	6 C+; 12 S+;<br>12 clin (16 pTB, 2 pTB/EPTB, 6 EPTB)
Kali[64]	2006	2003	South Africa	545	370*			1					2	2	2.12%	8 S-/C+
Nachega[65]	2003	2001	South Africa	438*	318			2		1 (5)		2	3(XXX)	3(XXX)	2.30%	6 C+; 5 S+; 2 clin (4 pTB, 9 EPTB)
High risk groups (MSM and IVDU)																
Khanani[101]	2008		Iran	700[c]	700[c]			1					2(XXX)		6.00%	

[a]The total number of VCT clients eligible for screening=1220 (no break-down according to HIV status), [b]Women were followed for a maximum of 12 months after giving birth 7 TB cases were found within the first 2 weeks of delivery, [c]300 IDUs and 200 MSMs (HIV positive and HIV negative) were screened

Hospital, clinic based, home based screening

Author	Year of Publication	Year of study	Country	Setting	$N_{(HIV+)}$ eligible	$N_{(HIV+)}$ screened	Symptom screen	Exam	TST	CXR	antibiotics	S	C	TB prevalence	Comments prevalence
Swaminathan[66]	2000	1989-93	India	STD clinic attendees	487	175*	1	1		1		2(XXXX)	2(XXXX)	9.71%	
Dhungana[67, 68]	2008	2004-05	Nepal	Out- and inpatients	100	100						1(SMS)	1(SMS)	7.00%	1 S+/C-; 4 S+/C+; 2 S-/C+
Khun[69]	2002	2001	Cambodia	Home-based	773	773	1	1		1		2	2	13.58%	29 S+; 29 S-/C+, 46 S-/C-/clin; 20 EPTB
Kimerling[70]	2002	2000	Cambodia	Home-based	787	441*						1(S)	1(S)	9.30%	29 S+/C+; 12 S-/C+
Ngowi[71]	2008	2006-07	Tanzania	HIV/ARV clinic	233	233						1(S)	1(S)	8.58%	8 S+/C+; 12 S-/C+
Bakari/Mtei[9, 72 a]	2008/2005	2001-03	Tanzania	HIV clinic	1176	1176	1			1		1(XXX)	1(XXX)	11.56%	2S+/C-; 13 S+/C+; 23 S-/C+; 98 S-/C-/clin
Nakanjako[73]	2007	2005-06	Uganda	HIV clinic	2469	2469	1			2		2 (XXX)		7.78%	
Were/Moore[74, 75]	2007/2009	2003-05	Uganda	HIV/ARV clinic	1995	1995	1			4	3	2 (SMS)	4	3.56%	14 S+; 53 S-/clin; 4EPTB
Gasana[76]	2008	2006	Rwanda	HIV clinic	629	300*	1			2		2		3.67%	
Bassett[77]	2009	2007-08	South Africa	ARV clinic	824	824	1					1	1	19.30%	14 S+/C+, 145 S-/C+
Lawn[10]	2009	2007-08	South Africa	ARV clinic	235	235	1					1(SS)	1 (SS)	24.68%	8 S+/C+, 50 S-/C+
Mohammed[78]	2004		South Africa	HIV clinic	140	129*	1			1		1(S)	1(S)	8.53%	6 S+/C+; 4S-/C+; 1 C-/clin
Reddy[79]	2008		Peru	HIV clinic	471	435*						1(XX)	1(XX)	6.21%	7 S+/C+, 20 S-/C+
Silva[80]	2004	2001-02	Brazil	Inpatients	547	547	1			1		2	2	1.83%	3 S+/C+; 7 S-/C+
Murcia[81]	2001	1999-00	Colombia	HIV clinic	286	286						1	1	1.40%	2 pTB; 2 EPTB
Crespo[82]	1999		Colombia	social service	155	155				1		2	2	6.45%	1 pTB; 6 miliary TB 2 TB meningitis; 1 lymph node TB

[a]inclusion criteria CD4 > 200

Contact tracing

Author	Year of Publication	Year of study	Country	$N_{(index)}$ eligible	$N_{(index)}$ included	$N_{(contact)}$ eligible	$N_{(contact)}$ screened	Symptom screen	Exam	TST	CXR	antibiotics	S	C	TB prevalence	Comments prevalence
Kuaban[83]	1996	1993-94	Cameroon	336	104	416	346*	1		1 (10)	1		2		14.45%	25 S+; 25 clin (25 cases in children <14)
Jackson-Sillah[84]	2007	2002-04	The Gambia	317	317	2381	2174*	1		1 (10)	2	4	3	3	1.52%	6 S+/C+; 18 S-/C+; 6 clin (16 cases in children <14)
Diatta[85]	2007	2003-04	Senegal	30	30	601	601	2	2	1 (8)	2		3		2.66%	16 S+ (2 cases in children<15)
Guwatudde[86]	2003	1995-99	Uganda	360	302	1206	1206	1	1		1		2(XXX)	2(XXX)	4.23%	13 S+/C+; 9 S-/C+; 9 S+/C-; 16 clin (34 cases in children<6)
Zachariah[87]	2003	2001-02	Malawi	87	87	461	461	1[a]					2(SM)		1.74%	2 S+; 6 clin (4 cases in children <6)
Suggaravetsiri[88]	2003	2000-02	Thailand	499	459	1200	1192	1	1	1 (15)	2		2(MMM)	3	3.75%	
Becerra[89]	2005	1997-98	Peru	208	208	1094	1094	1					2(MM)	2(MM)	0.70%	
Bayona[90]	2003	1996-99	Peru	192	192	945	945	1					2		0.01%	
Carvalho[91]	2003	1995-97	Brazil	191	86	371	360*	1		1(10/5)[b]	1		2	2	0.83%	
Teixeira[92]	2001	1994-98	Brazil	78[c]	78	408*	364	1	1	1 (10)	2		2	2	4.17%	

[a]children were screened with CXR followed by antibiotics, [b]cut-offs for HIV- individuals 10mm, cut off HIV+ individuals 5mm, [c]26 of the index cases had MDR TB

Population based

Author	Year of Publication	Year of study	Country	N eligible	N screened	Symptom screen	Exam	TST	CXR	antibiotics	S	C	TB prevalence	Comments prevalence
Demissie[93]	2002	2001	Ethiopia	12149	12149	1					2(SMS)		0.17%	21 S+
Sekandi[94]	2009	2005	Uganda	1000	930	1					2(SMM)		3.50%	33 S+
Guwatudde[95]	2003	2001-02	Uganda	1142	1142	1			2		2(SMM)	2(SMM)	0.70%	
Corbett[96]	2007		Zimbabwe	10079	10079						1(SM)	1(SM)	0.65%	40 S+/C+, 26 S-/C+
Corbett[97]	2007	2001	Zimbabwe	4884	4668*	1			3		2(XXX)	2(XXX)	0.58%	6 S+/C+; 15 S-/C+; 6 clin
den Boon[98, 99]	2006/2007	2002	South Africa	3971	2608*				1		1(S)	1(S)	1.11%	20 S+/C+; 7 S-/C+; 2 scanty smear
Wood[11]	2007	2005	South Africa	971	762*						1(MS)	1(MS)	1.57%	6 S+/C+; 6 S-/C+
Pronyk[100]	2001	1999	South Africa	38251*	38127	1					2(SM)	2(X)	0.02%	6 S+/C+

TST=Tuberculosis skin test, CXR= chest radiograph, S=sputum smear, C=sputum culture, clin=clinical, EPTP=extrapulmonary TB, pTB=pulmonary TB

Numbers indicate the order in which investigational screening tools were used in the screening process. For example the prison study by Aerts et al. performed symptom screening first indicated by a “1” in the symptom column. Individuals with symptoms had a CXR and sputum smears indicated by a “2” in these columns. All positive sputum smears were cultured indicated by a “3”.

Numbers in brackets in TST column indicate the cut-off for TST positivity. For example the study by Askarian et al. used a TST cut-off of 10mm.

A number in brackets in the CXR column indicates that CXRs were not performed on everybody (see prison study by Aerts et al.)

The number of letters in the smear and culture columns indicates the number of smears/cultures examined. “M” indicates early morning sputum, “S” indicates spot sputum and “X” indicates that the time when sputum was taken was unknown. In the prison study by Aerts et al. sputum smear examination were performed on 3 early morning sputum samples indicated by three “Ms”. Studies without alphabetical letters did not report the number or timing of sputum samples.

References

9. Mtei L, Matee M, Herfort O, Bakari M, Horsburgh CR, Waddell R, et al. High rates of clinical and subclinical tuberculosis among HIV-infected ambulatory subjects in Tanzania. Clin Infect Dis. 2005 May 15;40(10):1500-7.

10. Lawn SD, Edwards SD, Kranzer K, Vogt M, Bekker LG, Wood R. Urine lipoarabinomannan assay for tuberculosis screening prior to ART: diagnostic yield and association with immune reconstitution disease. Aids, in press. 2009.

11. Wood R, Middelkoop K, Myer L, Grant AD, Whitelaw A, Lawn SD, et al. Undiagnosed tuberculosis in a community with high HIV prevalence: implications for tuberculosis control. Am J Respir Crit Care Med. 2007 Jan 1;175(1):87-93.

22. Aerts A, Habouzit M, Mschiladze L, Malakmadze N, Sadradze N, Menteshashvili O, et al. Pulmonary tuberculosis in prisons of the ex-USSR state Georgia: results of a nation-wide prevalence survey among sentenced inmates. Int J Tuberc Lung Dis. 2000 Dec;4(12):1104-10.

23. Salek S, Taghizadeh AR, Yazdanpanah M, Masjedi M, Velayati AA, Mohammadi HR, et al. Case finding of pulmonary tuberculosis in Ghasr prison. 32th World Conference on Lung Health of the IUATL Paris; 2001; November 1-4.

24. Askarian M, Karmi A, Sadeghi-Hassanabadi A. Tuberculosis among never-jailed drug abusers. East Mediterr Health J. 2001 May;7(3):461-4.

25. Yazdanpanah M. To assess the pulmonary tuberculosis among prisoners. 30th World Conference on Lung Health of the IUATL Madrid; 1999; September 14-18.

26. Rao NA. Prevalence of pulmonary tuberculosis in Karachi central prison. J Pak Med Assoc. 2004 Aug;54(8):413-5.

27. Shah SA, Mujeeb SA, Mirza A, Nabi KG, Siddiqui Q. Prevalence of pulmonary tuberculosis in Karachi juvenile jail, Pakistan. East Mediterr Health J. 2003 Jul;9(4):667-74.

28. Noeske J, Kuaban C, Amougou G, Piubello A, Pouillot R. Pulmonary tuberculosis in the Central Prison of Douala, Cameroon. East Afr Med J. 2006 Jan;83(1):25-30.

29. Koffi N, Ngom AK, Aka-Danguy E, Seka A, Akoto A, Fadiga D. Smear positive pulmonary tuberculosis in a prison setting: experience in the penal camp of Bouake, Ivory Coast. Int J Tuberc Lung Dis. 1997 Jun;1(3):250-3.

30. Rapid assessment of tuberculosis in a large prison system--Botswana, 2002. MMWR Morb Mortal Wkly Rep. 2003 Mar 28;52(12):250-2.

31. Banerjee A, Harries AD, Mphasa N, Yadid AE, Nyirenda T, Salaniponi FM. Prevalence of HIV, sexually transmitted disease and tuberculosis amongst new prisoners in a district prison, Malawi. Trop Doct. 2000 Jan;30(1):49-50.

32. Nyangulu DS, Harries AD, Kang'ombe C, Yadidi AE, Chokani K, Cullinan T, et al. Tuberculosis in a prison population in Malawi. Lancet. 1997 Nov 1;350(9087):1284-7.

33. Habeenzu C, Mitarai S, Lubasi D, Mudenda V, Kantenga T, Mwansa J, et al. Tuberculosis and multidrug resistance in Zambian prisons, 2000-2001. Int J Tuberc Lung Dis. 2007 Nov;11(11):1216-20.

34. Pillay M, Govender K, Reddy S, Roux L, Kagoro H, Sturm AW. The prevalence of tuberculosis in a South African prison. 34th World Conference on Lung Health of the IUATL Paris; 2003; October 29 - November 2.

35. Naranbat N, Otgontsetseg D, Nymadawa P, Tsogt G. Tuberculosis in Mongolian prisons. 32th World Conference on Lung Health of the IUATL Paris; 2001; November 1-4.
36. Jittimanee SX, Ngamtrairai N, White MC, Jittimanee S. A prevalence survey for smear-positive tuberculosis in Thai prisons. Int J Tuberc Lung Dis. 2007 May;11(5):556-61.
37. Sretrirutchai S, Silapapojakul K, Palittapongarnpim P, Phongdara A, Vuddhakul V. Tuberculosis in Thai prisons: magnitude, transmission and drug susceptibility. Int J Tuberc Lung Dis. 2002 Mar;6(3):208-14.
38. Sanchez AR, Massari V, Gerhardt G, Barreto AW, Cesconi V, Pires J, et al. [Tuberculosis in Rio de Janeiro prisons, Brazil: an urgent public health problem]. Cad Saude Publica. 2007 Mar;23(3):545-52.
39. Abrahao RM, Nogueira PA, Malucelli MI. Tuberculosis in county jail prisoners in the western sector of the city of Sao Paulo, brazil. Int J Tuberc Lung Dis. 2006 Feb;10(2):203-8.
40. Fournet N, Sanchez A, Massari V, Penna L, Natal S, Biondi E, et al. Development and evaluation of tuberculosis screening scores in Brazilian prisons. Public Health. 2006 Oct;120(10):976-83.
41. Sanchez A, Gerhardt G, Natal S, Capone D, Espinola A, Costa W, et al. Prevalence of pulmonary tuberculosis and comparative evaluation of screening strategies in a Brazilian prison. Int J Tuberc Lung Dis. 2005 Jun;9(6):633-9.
42. Ferreira MM, Ferrazoli L, Palaci M, Salles PS, Medeiros LA, Novoa P, et al. Tuberculosis and HIV infection among female inmates in Sao Paulo, Brazil: a prospective cohort study. J Acquir Immune Defic Syndr Hum Retrovirol. 1996 Oct 1;13(2):177-83.
43. Van Duc L, Vree M, Cobelens FG, Phuc LT, Sy DN. High tuberculosis prevalence in a psychiatric hospital in Vietnam. Int J Tuberc Lung Dis. 2008 Jun;12(6):686-8.
44. Steen TW, Gyi KM, White NW, Gabosianelwe T, Ludick S, Mazonde GN, et al. Prevalence of occupational lung disease among Botswana men formerly employed in the South African mining industry. Occup Environ Med. 1997 Jan;54(1):19-26.
45. Fielding K, Chihota V, lewis J, Luttig M, Crawford T, Popane F, et al. Factors associated with prevalent TB at screening prior to isoniazid preventive therapy. 39th World Conference on Lung Health of the IUATL Paris; 2008; October 16-20.
46. Girdler-Brown BV, White NW, Ehrlich RI, Churchyard GJ. The burden of silicosis, pulmonary tuberculosis and COPD among former Basotho goldminers. Am J Ind Med. 2008 Sep;51(9):640-7.
47. Day JH, Charalambous S, Fielding KL, Hayes RJ, Churchyard GJ, Grant AD. Screening for tuberculosis prior to isoniazid preventive therapy among HIV-infected gold miners in South Africa. Int J Tuberc Lung Dis. 2006 May;10(5):523-9.
48. Corbett EL, Charalambous S, Moloi VM, Fielding K, Grant AD, Dye C, et al. Human immunodeficiency virus and the prevalence of undiagnosed tuberculosis in African gold miners. Am J Respir Crit Care Med. 2004 Sep 15;170(6):673-9.
49. Aungkasuvapala N, Juengprasert W, Obhasi N. Silicosis and pulmonary tuberculosis in stone-grinding factories in Saraburi, Thailand. J Med Assoc Thai. 1995 Dec;78(12):662-9.
50. Weinstock DM, Hahn O, Wittkamp M, Sepkowitz KA, Khechinashvili G, Blumberg HM. Risk for tuberculosis infection among internally displaced persons in the Republic of Georgia. Int J Tuberc Lung Dis. 2001 Feb;5(2):164-9.
51. Nelson LJ, Davis AB, McCrann CH, Sugo CP, Sanoto R, Notha M, et al. Tuberculosis screening at a refugee camp in Botswana 2002-2003. 34th World Conference on Lung Health of the IUATL Paris; 2003; October 29 - November 2.
52. Shetty PV, Granich RM, Patil AB, Sawant SK, Sahu S, Wares DF, et al. Cross-referral between voluntary HIV counselling and testing centres and TB services, Maharashtra, India, 2003-2004. Int J Tuberc Lung Dis. 2008 Mar;12(3 Suppl 1):26-31.
53. Shah S, Demissie M, Lambert L, Ahmed J, Leulseged S, Kebede T, et al. Intensified tuberculosis case finding among HIV-Infected persons from a voluntary counseling and testing center in Addis Ababa, Ethiopia. J Acquir Immune Defic Syndr. 2009 Apr 15;50(5):537-45.
54. Mugisha B, Bock N, Mermin J, Odeke RM, Miller B, Adatu-Engwau F, et al. Tuberculosis case finding and preventive therapy in an HIV voluntary counseling and testing center in Uganda. Int J Tuberc Lung Dis. 2006 Jul;10(7):761-7.
55. Aisu T, Raviglione MC, van Praag E, Eriki P, Narain JP, Barugahare L, et al. Preventive chemotherapy for HIV-associated tuberculosis in Uganda: an operational assessment at a voluntary counselling and testing centre. Aids. 1995 Mar;9(3):267-73.
56. Godfrey-Faussett P, Baggaley R, Mwinga A, Hosp M, Porter J, Luo N, et al. Recruitment to a trial of tuberculosis preventive therapy from a voluntary HIV testing centre in Lusaka: relevance to implementation. Trans R Soc Trop Med Hyg. 1995 Jul-Aug;89(4):354-8.
57. Naidoo P, Karpakis B, Maartens G, Schoenman H, Hausler HP. Active tubeculosis case-finding and isoniazid preventive therapy in HIV-positive clients at voluntary counselling and testing centres. 14th International AIDS Conference; Barcelona; 2002; July 7-12.

58. Kanara N, Cain KP, Laserson KF, Vannarith C, Sameourn K, Samnang K, et al. Using program evaluation to improve the performance of a TB-HIV project in Banteay Meanchey, Cambodia. Int J Tuberc Lung Dis. 2008 Mar;12(3 Suppl 1):44-50.
59. Screening HIV-infected persons for tuberculosis--Cambodia, January 2004-February 2005. MMWR Morb Mortal Wkly Rep. 2005 Nov 25;54(46):1177-80.
60. Chheng P, Tamhane A, Natpratan C, Tan V, Lay V, Sar B, et al. Pulmonary tuberculosis among patients visiting a voluntary confidential counseling and testing center, Cambodia. Int J Tuberc Lung Dis. 2008 Mar;12(3 Suppl 1):54-62.
61. Espinal MA, Reingold AL, Koenig E, Lavandera M, Sanchez S. Screening for active tuberculosis in HIV testing centre. Lancet. 1995 Apr 8;345(8954):890-3.
62. Burgess AL, Fitzgerald DW, Severe P, Joseph P, Noel E, Rastogi N, et al. Integration of tuberculosis screening at an HIV voluntary counselling and testing centre in Haiti. Aids. 2001 Sep 28;15(14):1875-9.
63. Gupta A, Nayak U, Ram M, Bhosale R, Patil S, Basavraj A, et al. Postpartum tuberculosis incidence and mortality among HIV-infected women and their infants in Pune, India, 2002-2005. Clin Infect Dis. 2007 Jul 15;45(2):241-9.
64. Kali PB, Gray GE, Violari A, Chaisson RE, McIntyre JA, Martinson NA. Combining PMTCT with active case finding for tuberculosis. J Acquir Immune Defic Syndr. 2006 Jul;42(3):379-81.
65. Nachega J, Coetzee J, Adendorff T, Msandiwa R, Gray GE, McIntyre JA, et al. Tuberculosis active case-finding in a mother-to-child HIV transmission prevention programme in Soweto, South Africa. Aids. 2003 Jun 13;17(9):1398-400.
66. Swaminathan S, Ramachandran R, Baskaran G, Paramasivan CN, Ramanathan U, Venkatesan P, et al. Risk of development of tuberculosis in HIV-infected patients. Int J Tuberc Lung Dis. 2000 Sep;4(9):839-44.
67. Dhungana GP, Ghimire P, Sharma S, Rijal BP. Characterization of mycobacteria in HIV/AIDS patients of Nepal. JNMA J Nepal Med Assoc. 2008 Jan-Mar;47(169):18-23.
68. Dhungana GP, Ghimire P, Sharma S, Rijal BP. Tuberculosis co-infection in HIV infected persons of Kathmandu. Nepal Med Coll J. 2008 Jun;10(2):96-9.
69. Khun KE, Tamura M, Yous BH, I. O, Mao TE. New TB screening service for poeple living with HIV/AIDS (PLWHA) in Phnom Penh. 33th World Conference on Lung Health of the IUATL Montréal; 2002; October 6-10.
70. Kimerling ME, Schuchter J, Chanthol E, Kunthy T, Stuer F, Glaziou P, et al. Prevalence of pulmonary tuberculosis among HIV-infected persons in a home care program in Phnom Penh, Cambodia. Int J Tuberc Lung Dis. 2002 Nov;6(11):988-94.
71. Ngowi BJ, Mfinanga SG, Bruun JN, Morkve O. Pulmonary tuberculosis among people living with HIV/AIDS attending care and treatment in rural northern Tanzania. BMC Public Health. 2008;8(341):341.
72. Bakari M, Arbeit RD, Mtei L, Lyimo J, Waddell R, Matee M, et al. Basis for treatment of tuberculosis among HIV-infected patients in Tanzania: the role of chest x-ray and sputum culture. BMC Infect Dis. 2008;8:32.
73. Nakanjako D, Mwesigire D, Wanyenze R, Sempira J, Ouma J, Senkusu J, et al. Reinforcement of TB screening identifies a high burden of pulmonary tuberculosis among HIV/AIDS patients in the Mulago Immune Suppression Clinic, Uganda. 4th IAS Conference; Sydney, Australia; 2007; July 22-25.
74. Moore D, Liechty C, Ekwaru P, Were W, Mwima G, Solberg P, et al. Prevalence, incidence and mortality associated with tuberculosis in HIV-infected patients initiating antiretroviral therapy in rural Uganda. Aids. 2007 Mar 30;21(6):713-9.
75. Were W, Moore D, Ekwaru P, Mwima G, Bunnell R, Kaharuza F, et al. A simple screening tool for active tuberculosis in HIV-infected adults receiving antiretroviral treatment in Uganda. Int J Tuberc Lung Dis. 2009 Jan;13(1):47-53.
76. Gasana M, Vandebriel G, Kabanda G, Tsiouris SJ, Justman J, Sahabo R, et al. Integrating tuberculosis and HIV care in rural Rwanda. Int J Tuberc Lung Dis. 2008 Mar;12(3 Suppl 1):39-43.
77. Bassett I, Chetty S, Wand B, Giddy J, Losina E, Mazibuko M, et al. Intensive TB screening for HIV-infected patients ready to start ART in Durban, South Africa: limitations of WHO guidelines. 16th Confernce on Retroviruses and Opportunistic Infections; Montreal; 2009; February 8-11.
78. Mohammed A, Ehrlich R, Wood R, Cilliers F, Maartens G. Screening for tuberculosis in adults with advanced HIV infection prior to preventive therapy. Int J Tuberc Lung Dis. 2004 Jun;8(6):792-5.
79. Reddy KP, Brady MF, Gilman RH, Coronel J, Navincopa M, Ticona E, et al. MODS for tuberculosis screening prior to isoniazid preventive therapy in HIV-infected persons. 39th World Conference on Lung Health of the IUATL Paris; 2008; October 16-20.
80. Silva RM, Teixeira PJ, Moreira JdS. Induced sputum for the diagnosis of lung disease in HIV-positive patients. Jornal Brasileiro de Pneumologia Sociedade Brasileira de Pneumologia Tisiologia, Sao Paulo, Brazil. 2004;30(5):452-8.
81. Murcia-Aranguren MI, Gomez-Marin JE, Alvarado FS, Bustillo JG, de Mendivelson E, Gomez B, et al. Frequency of tuberculous and non-tuberculous mycobacteria in HIV infected patients from Bogota, Colombia. BMC Infect Dis. 2001;1(21):21.

82. Crespo MP, Heli Corral R, Alzate A, Carrasquilla G, Sanchez N. [Mycobacterial infections in HIV-infected patients in Cali, Colombia]. Rev Panam Salud Publica. 1999 Oct;6(4):249-55.
83. Kuaban C, Koulla-Shiro S, Lekama Assiene T, Hagbe P. [Tuberculosis screening of patient contacts in 1993 and 1994 in Yaounde, Cameroon]. Med Trop (Mars). 1996;56(2):156-8.
84. Jackson-Sillah D, Hill PC, Fox A, Brookes RH, Donkor SA, Lugos MD, et al. Screening for tuberculosis among 2381 household contacts of sputum-smear-positive cases in The Gambia. Trans R Soc Trop Med Hyg. 2007 Jun;101(6):594-601.
85. Diatta A, Toure NO, Kane YD, Ndiaye EH, Niang A, Thiam K, et al. [Familial tuberculosis: tracing the contacts of an infectious case]. Rev Mal Respir. 2007 Jan;24(1):32-40.
86. Guwatudde D, Nakakeeto M, Jones-Lopez EC, Maganda A, Chiunda A, Mugerwa RD, et al. Tuberculosis in household contacts of infectious cases in Kampala, Uganda. Am J Epidemiol. 2003 Nov 1;158(9):887-98.
87. Zachariah R, Spielmann MP, Harries AD, Gomani P, Graham SM, Bakali E, et al. Passive versus active tuberculosis case finding and isoniazid preventive therapy among household contacts in a rural district of Malawi. Int J Tuberc Lung Dis. 2003 Nov;7(11):1033-9.
88. Suggaravetsiri P, Yanai H, Chongsuvivatwong V, Naimpasan O, Akarasewi P. Integrated counseling and screening for tuberculosis and HIV among household contacts of tuberculosis patients in an endemic area of HIV infection: Chiang Rai, Thailand. Int J Tuberc Lung Dis. 2003 Dec;7(12 Suppl 3):S424-31.
89. Becerra MC, Pachao-Torreblanca IF, Bayona J, Celi R, Shin SS, Kim JY, et al. Expanding tuberculosis case detection by screening household contacts. Public Health Rep. 2005 May-Jun;120(3):271-7.
90. Bayona J, Chavez-Pachas AM, Palacios E, Llaro K, Sapag R, Becerra MC. Contact investigations as a means of detection and timely treatment of persons with infectious multidrug-resistant tuberculosis. Int J Tuberc Lung Dis. 2003 Dec;7(12 Suppl 3):S501-9.
91. Carvalho AC, DeRiemer K, Nunes ZB, Martins M, Comelli M, Marinoni A, et al. Transmission of Mycobacterium tuberculosis to contacts of HIV-infected tuberculosis patients. Am J Respir Crit Care Med. 2001 Dec 15;164(12):2166-71.
92. Teixeira L, Perkins MD, Johnson JL, Keller R, Palaci M, do Valle Dettoni V, et al. Infection and disease among household contacts of patients with multidrug-resistant tuberculosis. Int J Tuberc Lung Dis. 2001 Apr;5(4):321-8.
93. Demissie M, Zenebere B, Berhane Y, Lindtjorn B. A rapid survey to determine the prevalence of smear-positive tuberculosis in Addis Ababa. Int J Tuberc Lung Dis. 2002 Jul;6(7):580-4.
94. Sekandi JN, Neuhauser D, Smyth K, Whalen CC. Active case finding of undetected tuberculosis among chronic coughers in a slum setting in Kampala, Uganda. Int J Tuberc Lung Dis. 2009 Apr;13(4):508-13.
95. Guwatudde D, Zalwango S, Kamya MR, Debanne SM, Diaz MI, Okwera A, et al. Burden of tuberculosis in Kampala, Uganda. Bull World Health Organ. 2003;81(11):799-805.
96. Corbett EL, Bandason T, Cheung YB, Makamure B, Dauya E, Matambo R, et al. Undiagnosed infectious tuberculosis in Harare, Zimbabwe: HIV, past TB treatment and other risk factors. 38th World Conference on Lung Health of the IUATL Cape Town 2007; November 8-12.
97. Corbett EL, Bandason T, Cheung YB, Munyati S, Godfrey-Faussett P, Hayes R, et al. Epidemiology of tuberculosis in a high HIV prevalence population provided with enhanced diagnosis of symptomatic disease. PLoS Med. 2007 Jan;4(1):e22.
98. den Boon S, van Lill SW, Borgdorff MW, Enarson DA, Verver S, Bateman ED, et al. High prevalence of tuberculosis in previously treated patients, Cape Town, South Africa. Emerg Infect Dis. 2007 Aug;13(8):1189-94.
99. den Boon S, White NW, van Lill SW, Borgdorff MW, Verver S, Lombard CJ, et al. An evaluation of symptom and chest radiographic screening in tuberculosis prevalence surveys. Int J Tuberc Lung Dis. 2006 Aug;10(8):876-82.
100. Pronyk PM, Joshi B, Hargreaves JR, Madonsela T, Collinson MA, Mokoena O, et al. Active case finding: understanding the burden of tuberculosis in rural South Africa. Int J Tuberc Lung Dis. 2001 Jul;5(7):611-8.
